# Comparative assessment of immunochromatographic test kits using low-molecular-weight antigens from cyst fluids of two different genotypes of *Taenia solium* for serodiagnosis of human cysticercosis

**DOI:** 10.1051/parasite/2026003

**Published:** 2026-01-23

**Authors:** Lakkhana Sadaow, Penchom Janwan, Patcharaporn Boonroumkaew, Rutchanee Rodpai, Oranuch Sanpool, Tongjit Thanchomnang, Marcello Otake Sato, Pewpan M. Intapan, Hiroshi Yamasaki, Yasuhito Sako, Toni Wandra, Kadek Swastika, Wanchai Maleewong

**Affiliations:** 1 Mekong Health Science Research Institute, Khon Kaen University Khon Kaen 40002 Thailand; 2 Department of Parasitology, Faculty of Medicine, Khon Kaen University Khon Kaen 40002 Thailand; 3 Department of Medical Technology, School of Allied Health Sciences, Walailak University Nakhon Si Thammarat 80161 Thailand; 4 Department of Medical Technology, Faculty of Allied Health Sciences, Nakhonratchasima College Nakhon Ratchasima 30000 Thailand; 5 Department of Parasitology, Faculty of Medicine, Mahasarakham University Maha Sarakham 44000 Thailand; 6 Division of Global Environment Parasitology, Faculty of Medical Technology, Niigata University of Pharmacy and Medical and Life SciencesNiigata 956-8603 Japan; 7 Department of Parasitology, National Institute of Infectious Diseases, Japan Institute for Health security Tokyo 162-8640 Japan; 8 Division of Parasitology, Department of Infectious Diseases, Asahikawa Medical University Asahikawa 078-8510 Japan; 9 Sari Mutiara Indonesia University Medan, North Sumatra Indonesia; 10 Department of Parasitology, Faculty of Medicine, Udayana University Denpasar, Bali Indonesia

**Keywords:** *Taenia solium*, Genotypes, Human cysticercosis, Immunochromatographic test, Antibody detection, Diagnostics

## Abstract

Human cysticercosis is a serious zoonosis caused by infection with larvae (cysticerci) of the pork tapeworm, *Taenia solium*. Infection can involve the nervous system, causing chronic headache and intracranial hypertension, focal neurological deficits, epileptic seizures, and paralysis. The disease is found in developing countries, where porcine cysticercosis is prevalent and undercooked pork is habitually consumed. This study aimed to develop immunochromatography-based test (ICT) kits, using low-molecular-weight antigens purified from cyst fluids of Latin American and Asian genotypes of *T. solium*. To evaluate the kits, we used 164 serum samples, including 24 from proven/confirmed cysticercosis cases, 110 from cases with other parasitoses, and 30 from healthy individuals. Diagnostic performances were calculated. The sensitivity, specificity, and accuracy were 83.3% (95% CI [62.6–95.3]), 93.6% (95% CI [88.1–97.0]), and 92.1% (95% CI [86.8–95.7]), respectively for the American genotype-based ICT kit, while for the Asian genotype-based ICT kit, they were 87.5% (95% CI [67.6–97.3]), 98.6% (95% CI [94.9–99.8]), and 97.0% (95% CI [93.0–99.0]), respectively. The sensitivity and specificity did not significantly differ between the two ICT kits (exact McNemar’s test; *p* > 0.05), with a concordance of 93.9%, represented by a Cohen’s kappa of 0.77 (*p* < 0.001), indicating substantial agreement. These results indicate that affinity-purified antigens from different geographical isolates can be used for the diagnosis of human cysticercosis. The diagnostic specificities were better than for a previously reported ICT kit that used crude antigen.

## Abstract (fr)

La cysticercose humaine est une zoonose grave causée par l’infection par les larves (cysticerques) du ténia du porc, *Taenia solium*. L’infection peut atteindre le système nerveux et provoquer des céphalées chroniques et une hypertension intracrânienne, des déficits neurologiques focaux, des crises d’épilepsie et une paralysie. Cette maladie est présente dans les pays en développement, où la cysticercose porcine est endémique et où la consommation de porc insuffisamment cuit est courante. Cette étude visait à développer des tests immunochromatographiques (TIC) utilisant des antigènes de faible poids moléculaire purifiés à partir de liquides kystiques de génotypes latino-américain et asiatique de *T. solium*. Pour évaluer les kits, nous avons utilisé 164 échantillons de sérum, dont 24 provenant de cas de cysticercose confirmés, 110 de cas d’autres parasitoses et 30 de sujets sains. Les performances diagnostiques ont été calculées. La sensibilité, la spécificité et la précision étaient respectivement de 83,3 % (IC à 95 % [62,6-95,3]), 93,6 % (IC à 95 % [88,1-97,0]) et 92,1 % (IC à 95 % [86,8-95,7]) pour le kit TIC basé sur le génotype américain, et de 87,5 % (IC à 95 % [67,6-97,3]), 98,6 % (IC à 95 % [94,9-99,8]) et 97,0 % (IC à 95 % [93,0-99,0]) pour le kit TIC basé sur le génotype asiatique. La sensibilité et la spécificité ne différaient pas significativement entre les deux kits TIC (test exact de McNemar; *p* > 0,05), avec une concordance de 93,9 %, représentée par un kappa de Cohen de 0,77 (*p* < 0,001), indiquant un accord substantiel. Ces résultats indiquent que les antigènes purifiés par affinité provenant d’isolats de différentes régions géographiques peuvent être utilisés pour le diagnostic de la cysticercose humaine. Les spécificités diagnostiques étaient supérieures à celles d’un kit TIC précédemment décrit utilisant un antigène brut.

## Introduction

Cysticercosis is a harmful food-borne zoonosis caused by infection with larvae (cysticerci) of the pork tapeworm, *Taenia solium*. Human cysticercosis occurs in developing countries where porcine cysticercosis is endemic and uncooked or semi-cooked pork is habitually consumed. Cysticercosis is primarily reported from Southeast and South Asia, Sub-Saharan Africa, and Central and South America [35]. Imported cysticercosis cases are sometimes found, due to international travel, in regions where cysticercosis is not endemic [27, 32].

The *T. solium* life cycle requires humans as the sole definitive host and pigs as the intermediate host [7]. Infection leading to adult worms in the human gut occurs when undercooked pork containing cysticerci is eaten. If *T. solium* eggs are ingested instead, oncospheres hatching from the eggs in the intestine migrate via the blood and lymph stream, and cysticerci develop in the central nervous system and systemic musculature [7]. Thus humans can also act as a dead-end intermediate host. Under some circumstances, eggs from adult worms in the human intestine can hatch in the host (autoinfection), releasing oncospheres that can invade tissues and become cysticerci [19].

Neurocysticercosis (NCC) occurs if the central nervous system is invaded by *T. solium* cysticerci. NCC often presents as neurological disorders such as epileptic seizures and paralysis [26]. Importantly, NCC is the main cause of epilepsy cases (30%) in areas where people and free-roaming pigs live in close proximity [35]. Subcutaneous cysticercosis (SCC), distinguished by unmoving nodule(s) in the musculature, including the extremities, and ocular or orbital cysticercosis, when the eyes are infested with cysticerci, are other forms of cysticercosis [17].

Accurate diagnosis of human cysticercosis is necessary for proper treatment and for prevention of severe clinical manifestations [24]. Diagnosis is normally based on neuroimaging using computed tomography and magnetic resonance imaging, as well as serology and pathology findings [3]. Various serodiagnostic tools, including commercial kits, for example ELISA and immunoblot assays, have been described [15]. These tools use antigens produced from crude or partially purified products of *T. solium* cyst fluid or cyst-tissue extracts [7]. Recombinant antigens [21] or peptide antigens [10] have also been used. Enzyme linked immunoelectrotransfer blot assay using lentil lectin purified parasite glycoprotein antigens showed sensitivity above 98% and specificity is approximate 100% [30]. These serological methods are time consuming and expensive, require complex equipment and infrastructure as well as trained technicians, and are not practical in resource-limited settings.

Various rapid tests have been reported for human cysticercosis diagnosis such as the magnetic immunochromatography test, quick ELISA, lateral flow assay, and latex agglutination test, and showed sensitivity of 52–96.3% and the specificity of 96–100% [15]. Commercial kits, for example the CYSTICERCOSIS Western Blot IgG kit^®^ (LDBIO Diagnostics, Lyon, France), are also available. Recently, an immunochromatography-based point-of-care test (POCT) kit “named the “iCysticercosis kit” was developed to detect anti-*T. solium* IgG antibodies in human serum samples [18]. This kit uses crude cyst fluid of *T. solium* from Brazil as the antigen source. The diagnostic values of the kit for sensitivity, specificity, and accuracy were 83.3%, 92.0%, and 90.9%, respectively. The iCysticercosis kit has sensitivity of 83.3% when tested in 21 NCC, 2 ocular cysticercosis, and one subcutaneous cysticercosis serum samples [18]. However, the iCysticercosis kit showed frequent cross-reactions when evaluated with sera from cases of cystic echinococcosis (10/30; 33.3%) and alveolar echinococcosis (1/6; 16.7%). Here, we developed two immunochromatographic test (ICT) kits using partially purified antigens, the immunodiagnostic low-molecular-weight antigens (LMWAgs), from cyst fluids of two different genotypes of *T. solium*: the Asian and Afro/American genotypes [22]. LMWAgs are glycoproteins and are part of a 150-kDa hydrophobic ligand-binding protein (HLBP) that may be involved in the uptake of fatty acids from the host for parasite survival [12]. In addition, LMWAgs have been demonstrated to provide a highly accurate serodiagnosis of cysticercosis, even though there are different sugar moieties between Asian and Afro/American genotypes, resulting in different antigenicity [20, 22, 23]. The diagnostic results obtained using these kits for the serodiagnosis of human cysticercosis were compared.

## Materials and methods

### Preparation of low-molecular-weight antigens (LMWAgs)

*Taenia solium* cysticerci were collected from necropsied pigs in Piauí State, Brazil (the American genotype) and in Bali, Indonesia (Asian genotype). Genotypes were confirmed based on the DNA sequence analysis of the PCR-amplified cytochrome *c* oxidase subunit I gene [22]. The partially purified antigens, which included LMWAgs, were prepared as previously reported [20]. Briefly, crude cyst fluid was extracted from individual cysticerci by aspiration using a 1-mL syringe. This cyst fluid was centrifuged, and the supernatant fluid was dialyzed against a start buffer (10 mM HEPES, 0.5 mM EDTA, pH 8.0). After adding CHAPS to the dialysate, up to a final concentration of 2%, it was directly loaded onto a HiTrap SP XL cation-exchange column (GE healthcare, Marlborough, MA, USA) pre-equilibrated with the start buffer. The column was then washed with the start buffer. Proteins were recovered manually by stepwise elution, with the start buffer containing 1.0 M NaCl. The eluate was boiled for 20 min to precipitate contaminants; then, the supernatant containing LMWAgs was collected and kept at −20 °C for use as antigen. The purified antigens were named B1 and I2 from Brazilian and Indonesian *T. solium*, respectively. The presence of immunogenic components of approximately 10 kDa was confirmed, consistent with previous reports [20] that identified immunogenic bands in the enzyme-linked immunoelectrotransfer blot (EITB) ranging from 10 to 25 kDa. The final protein concentration of each antigen preparation was estimated using a Pierce BCA Protein Assay kit (Thermo Fisher Scientific, Pleasanton, CA, USA).

### Human sera

Frozen leftover serum samples (*n* = 164), which had been stored at the serum bank, Faculty of Medicine and Mekong Health Science Research Institute Biobank project, Khon Kaen University, Thailand and Department of Parasitology, National Institute of Infectious Diseases, Tokyo, Japan, were used for comparative assessment of the kits (Table 1). Since the earliest serum sample dates back to 1987, storage times at −70 °C range from 10 to 38 years. Almost all of these sera were from Asian individuals (Supplementary Table 1). Demographic information and diagnostic criteria relating to the *T. solium* cysticercosis patients (*n* = 24) examined in the present study were previously described [18] and proven cysticercosis was diagnosed based on various criteria [3, 8], including clinical signs, CT scan, MRI and ultrasonography, serological (immunoblotting, LDBIO Diagnostics) and histopathological examinations, and/or molecular analysis [18]. Serum samples from cases other than cysticercosis examined have also been described previously [18], except for loiasis (*n* = 2) and anisakiasis (*n* = 5) [33]. Briefly, the sera used were as follows: from healthy persons who were free from any intestinal helminthic infection by stool examination and/or serologically negative against any parasitic infections (10 Thai and 20 Japanese individuals); sparganosis (*n* = 12, including 1 cerebral sparganosis); cystic echinococcosis (*n* = 28) and alveolar echinococcosis (*n* = 6); Taeniasis (*Taenia saginata*) (*n* = 5); angiostrongyliasis with eosinophilic meningoencephalitis (*n* = 10); gnathostomiasis (*n* = 5); toxocariasis (*n* = 2); loiasis (*n* = 2); anisakiasis (*n* = 5); trichinosis (*n* = 5); fascioliasis (*Fasciola gigantica*) (*n* = 10); paragonimiasis (*n* = 10, including an ectopic cerebral paragonimiasis case due to *Paragonimus westermani*); capillariasis (*Capillaria philippinensis*) (*n* = 5); and amebic liver abscess (*n* = 5) (Table 1).

**Table 1:** Result of the American genotype based-ICT (Am-ICT) and the Asian genotype based-ICT (As-ICT) kits for detection of anti-*Taenia solium* cysticerci IgG antibody in human samples.

Categories of serum sample	Number of positive cases /total number examined (range of test band intensity levels)	
	Am-ICT kit	As-ICT kit
Healthy persons	0/30 (0)	0/30 (0)
Cysticercosis (*Taenia solium*)	20/24 (1–5)	21/24 (0.5–8)
Sparganosis	0/12 (0)	0/12 (0)
Cystic echinococcosis	4/28 (0.5–1)	1/28 (3)
Alveolar echinococcosis	1/6 (2)	0/6 (0)
Taeniasis (*Taenia saginata*)	0/5 (0)	0/5 (0)
Angiostrongyliasis	0/10 (0)	0/10 (0)
Gnathostomiasis	0/5 (0)	0/5 (0)
Toxocariasis	1/2 (0.5)	1/2 (0.5)
Trichinosis	0/5 (0)	0/5 (0)
Capillariasis (*Capillaria philippinensis*)	0/5 (0)	0/5 (0)
Fascioliasis (*Fasciola gigantica*)	1/10 (2)	0/10 (0)
Paragonimiasis	0/10 (0)	0/10 (0)
Amoebiasis	0/5 (0)	0/5 (0)
Loiasis	2/2 (0.5–3)	0/2 (0)
Anisakiasis	0/5 (0)	0/5 (0)
Sensitivity (%)	83.3 (20/24) (95% CI [62.6–95.3])	87.5 (21/24) (95% CI [67.6–97.3])
Specificity (%)	93.6 (131/140) (95% CI [88.1–97.0])	98.6 (138/140) (95% CI [94.9–99.8])
Positive likelihood ratio	13.0 (95% CI [6.72–25.0])	61.3 (95% CI [15.3–245])
Negative likelihood ratio	0.178 (95% CI [0.0727 – 0.436])	0.127 (95% CI [0.044–0.366])
Accuracy (%)	92.1 (151/164) (95% CI [86.8–95.7])	97.0 (159/164) (95% CI [93.0–99.0])

The study was conducted in accordance with the Declaration of Helsinki and was approved by the Committee of the Center for Ethics in Human Research at Khon Kaen University (HE664044) and the Medical Ethics Committee of the National Institute of Infectious Diseases, Tokyo, Japan (Nos. 177, 589). The Human Ethics Committee waived the need for informed consent. We identified all samples by code, and they were thus fully anonymized.

### Immunochromatographic test (ICT) kits

For production of the ICT kits, LMWAgs, B1 and I2, from cyst fluids of American and Asian genotypes, respectively were used to detect total IgG antibody. The elements of the kit were as follows: sample pad (Kestrel BioSciences Co., Pathumthani, Thailand), conjugate-release pad (glass microfiber filter GF33; Whatman Schleicher & Schuell, Dassel, Germany), nitrocellulose membrane (Sartorius Stedim Biotech SA, Göttingen, Germany) on which were sprayed the test (T) and control (C) lines, absorbent pad (Kestrel BioSciences Co.), backing material (Kestrel BioSciences Co.), and cassette (Adtec Inc., Oita, Japan). The T line consisted of 1 mg/mL LMWAg from *T. solium* B1 or I2, and the C line contained goat anti-mouse IgG (1 mg/mL; 0.1 μL/mm) (Lampire Biological Laboratories, Pipersville, PA, USA). The conjugate release pad was injected with colloidal gold-conjugated mouse monoclonal anti-human IgG (Kestrel BioSciences Co., Ltd.). The spraying procedure was performed using an XYZ3210 Dispense Platform (BioDot, Irvine, CA, USA). The strip components were usually attached via the sticky backing material. The completed kit was placed in a resealable bag with a desiccant for storage at 4 °C.

**Figure 1:**
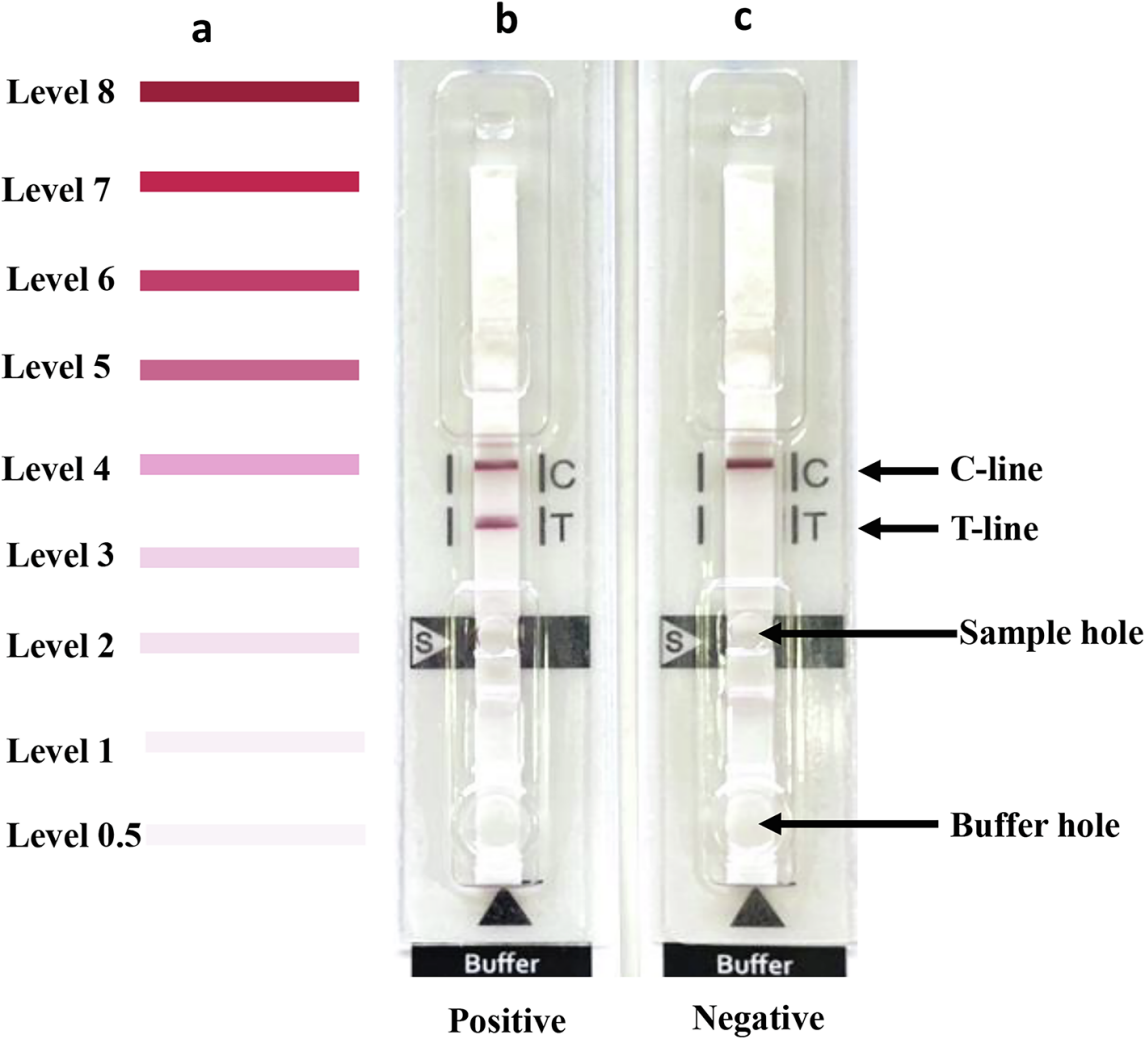
Immunochromatography test developed in this study. Card for interpretation of color intensity (levels 0.5–8) (a); positive case with red bands at both the control (C) and test (T) lines (b); negative case with a red band only at the C line (c).

To use either the Am-ICT kit or the As-ICT kit, the serum sample was mixed (1:15) with chromatography buffer (25 mM Tris-HCl, pH 8.0, and 0.25% casein); 5 μL of diluted serum was added into the serum (S) hole, and 60 μL of chromatography buffer was added into the buffer hole (Fig. 1). Interpretation of the result was based on the appearance of one or two red bands after 15 min. The intensity of any positive band (T line) was examined visually by comparison with the reference color card (the minimum cut-off level was 0.5) (Fig. 1). Blind samples were tested in duplicate, and two independent authors interpreted the results. The findings remained consistent even after repeated testing.

The diagnostic values were calculated as previously described [6], and Stata Statistical Software: Release 10 (StataCrop LP, College Station, TX, USA) was used to perform the sensitivity, specificity, positive and negative likelihood ratios, and Kappa value of this study. The sensitivity, specificity, and cross-reactivity of the two kits were compared using McNemar’s test. The total concordance was calculated using Cohen’s kappa test. Interpretations of kappa values (κ) were graded [13].

This study used the criteria of the STARD 2015 list (Supplementary Table 2) for reporting diagnostic accuracy [4].

## Results

**Figure 2:**
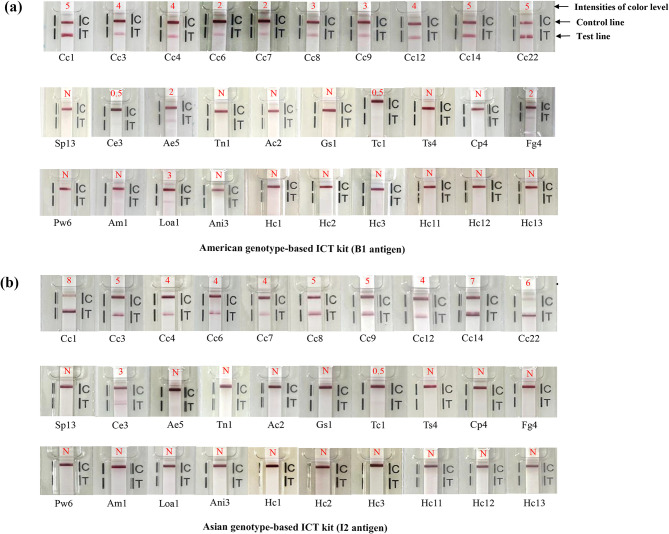
Representative results using the American (a) and Asian (b) genotype-based ICT kits. Cc1–Cc22, cysticercosis; Sp13, sparganosis; Ce3, cystic echinococcosis; Ae5, alveolar echinococcosis; Tn1, taeniasis (*Taenia saginata*); Ac2, angiostrongyliasis; Gs1, gnathostomiasis; Tc1, toxocariasis; Ts4, trichinosis; Cp4, capillariasis (*Capillaria philippinensis*); Fg4, Fascioliasis (*Fasciola gigantica*); Pw6, paragonimiasis (*Paragonimus westermani*); Am1, amebiasis; Loa1, loiasis; Ani3, Anisakiasis; Hc1–3; and Hc11–13, healthy Thai and Japanese controls. The color-intensity levels (0.5–8) are shown in each figure. N indicates negative results.

Serum samples from 24 cysticercosis cases consisting of NCC (racemose and multiple, *n* = 3), NCC (solitary, *n* = 2), NCC and SCC (multiple, *n* = 7), SCC (solitary, viable cyst, *n* = 1), NCC (multiple, *n* = 8), ocular type (*n* = 2), and NCC (type unknown, *n* = 1), 30 healthy persons and 110 other parasitosis cases were evaluated. The results for both ICT kits are summarized in Table 1, Figures 1 and 2, and Supplementary Table 1. Thirty healthy human controls all showed negative results with both ICT kits, while 20 cysticercosis cases were positive with the Am-ICT kit, and 21 cases were positive according to the As-ICT kit. A solitary case (Cc10) and ocular (Cc17) and multiple cases (Cc13, 15, 16) were negative in either Am-ICT or As-ICT kit, or both kits (Supplementary Table 1). Color intensity of the test band differed among cysticercosis cases (Table 1 and Supplementary Table 1). The As-ICT kit generally yielded more intense red-colored test bands (Supplementary Table 1). Cross-reactions were found in one cystic echinococcosis case and one toxocariasis case when using the As-ICT kit, while four cystic echinococcosis, one alveolar echinococcosis, one toxocariasis, one fascioliasis (*F. gigantica*), and two loiasis cases exhibited cross-reactions in the Am-ICT kit (Table 1). The sensitivity, specificity, and accuracy for the Am-ICT kit were 83.3% (20/24) (95% CI [62.6–95.3], 93.6% (131/140) (95% CI [88.1–97.0]), and 92.1% (151/164) (95% CI [86.8–95.7]), respectively, and 87.5% (21/24) (95% CI [67.6–97.3]), 98.6% (138/140) (95% CI [94.9–99.8]), and 97.0% (159/164) (95% CI [93.0–99.0]), respectively for the As-ICT kit (Table 1). Sensitivity and specificity did not differ significantly between the two test kits (exact McNemar’s test; *p* > 0.05), with a concordance of 93.9%, represented by a Cohen’s kappa of 0.77 (*p* < 0.001), indicating substantial agreement (*k* = 0.61–0.80).

## Discussion

A well-standardized immunodiagnostic test, the EITB assay using lentil lectin-purified glycoprotein extracts, is the test of choice for the specific detection of antibodies against *T. solium* antigens in serum or cerebrospinal fluid [8]. This EITB has specificity approaching 100% and sensitivity of 98% for individuals with two or more viable or degenerating parasites [30]. However, in patients with nonviable CNS infections, antibody responses may be diminished, thereby reducing diagnostic capability [1, 2, 9].

Currently, highly sensitive and specific rapid serodiagnostic tests for human cysticercosis, such as the magnetic immunochromatography test, quick ELISA, and lateral flow assay, are available using recombinant proteins [15, 16, 29]. These previous antibody detection tools are within the criteria for minimum performance requirements for cysticercosis diagnosis in terms of sensitivity and specificity proposed by the World Health Organization in the target product profiles for human cysticercosis [5]. Recently, the lateral flow POCT, the “TS POC test”, revealed 17% positive results for cysticercosis in rural southern Tanzania; however, the test yielded promising results for the diagnosis of NCC in patients with vesicular lesions [28]. The TS POC test is a two-strip lateral flow assay using the recombinant antigen rES33 on the TS POC T test strip, and rT24H on the TS POC CC test strip, to detect antibodies against *T. solium* taeniosis and cysticercosis, respectively [28, 31]. Van Damme *et al.* [31] evaluated the TS POC test at district-hospital level in Tanzania and found that, although its sensitivity was low, its specificity for both taeniosis and cysticercosis was high.

While previous native *T. solium* cyst-fluid antigen (American genotype from Brazil) [18] was used in the POCT kit (the iCysticercosis kit) and showed good sensitivity (83.3%) and specificity (92.0%) for cysticercosis, the test yielded high numbers of cross-reactions with human cystic echinococcosis (33.3%) and alveolar echinococcosis (16.7%) [18]. Variations in the diagnostic values between the present study and the previous reports might reflect the different conditions applied during ICT kit optimization, types of antigens and antibodies detected, and differences in the panels of samples used.

In this study, to limit these cross-reactivities, we developed new ICT kits using LMWAgs, B1 and I2, purified from cyst fluids of American and Asian genotypes of *T. solium*, respectively and evaluated their diagnostic values. The LMWAgs gave high immunodiagnostic performance from *T. solium* cyst fluids and are highly specific and sensitive for differential serodiagnosis of NCC in immunoblotting and/or an ELISA [11]. Our approach was informed by the observation that the cross-reactivities with echinococcosis observed in the ICT-based serodiagnosis using *T. solium* cyst fluid [18] are reduced by using cation-exchange chromatography-purified LMWAgs, without a decrease in sensitivity for cysticercosis. This result, together with the previously reported ELISA data, showed that cross-reactivity with sera from echinococcosis patients was eliminated [20]. As a result, the present ICT kits with sensitivities of 83.3–87.5%, matched those of the previous iCysticercosis kit [18], but with markedly reduced cross-reactivity. In our earlier report [18], cross-reactivity was 33% for cystic echinococcosis and 16.7% for alveolar echinococcosis. With the new kits, those cross-reactivity rates fell to 3.6% (As-ICT) and 14.3% (Am-ICT) for cystic echinococcosis, and to 0% (As-ICT) and 16.7% (Am-ICT) for alveolar echinococcosis.

In addition, we developed two types of ICT kit (As-ICT and Am-ICT) using LMWAgs (Afro/American and Asian types) partially purified from *T. solium* cysts isolated in Brazil and Indonesia, respectively to compare the serodiagnostic performance of both ICT kits. This was done because different antigenicity of a low molecular-weight hydrophilic protein family in cyst fluid purified between two genotypes of *T. solium* has been reported [23], indicating the possibility the source of LMWAgs affects serodiagnostic performance. This reason is supported by the reacted band intensity levels with the As-ICT kit (0.5–8), which were higher than the Am-ICT kit (1–5). However, there was no statistically significant difference in diagnostic values of both ICT kits according to the exact McNemar’s test (*p* > 0.05), with a concordance of 93.9% represented by a Cohen’s kappa of 0.77 (*p* < 0.001), indicating substantial agreement. Of the 24 cysticercosis sera used in this evaluation, only two were from patients in Africa (Malawi) and Latin America (Brazil), others were from Asian patients. Although it is difficult to conclude with certainty, because the number of specimens examined is too small, the intensity of the bands seems to tend to be stronger in multiple cysticercosis than in solitary, or ocular cysticercosis, as reported previously [34]. Therefore, further evaluation using sera from patients infected with the *T. solium* Afro/American genotype is needed.

Importantly, no false-positive reactions were observed with cases of parasitic diseases that require differential diagnosis from cysticercosis, e.g., cerebral sparganosis, paragonimiasis, and amebiasis. However, some cystic echinococcosis sera showed cross-reactions in both ICT kits, while one out of six alveolar echinococcosis sera showed a cross-reaction with the Am-ICT kit. This could be because echinococcosis patients, especially cystic echinococcosis patients, produce antibodies to *Echinococcus* Antigen B, which belongs to the same protein family as LMWAgs of *T. solium* [14, 25], and these antibodies show cross-reactivity to LMWAgs.

The kits are an easy-to-handle tool, useful not only for supporting clinical diagnosis at the bedside, but also for large-scale sero-epidemiological surveys in remote endemic areas where medical facilities or ancillary supplies are lacking. However, clinicians and laboratory technologists should be aware of the limitations of this study. Cross-reactions can occur with some echinococcosis, toxocariasis, fascioliasis (*F. gigantica*), and loiasis cases. Our evaluations were done in laboratory conditions using a circumscribed set of sera samples, with no information on *T. solium* cyst viability and stage of neurocysticercosis in cysticercosis cases and purified native antigen. To improve this tool, highly sensitive and specific recombinant antigens should be developed, and the performance of the tests still needs to be determined in a real-world setting.

## Conclusion

The present work proposes two types of ICT kits using purified antigens derived from different genotypes of *T. solium*. The kits showed no statistically significant difference in diagnostic values for the diagnosis of *T. solium* cysticercosis. These diagnostic values are better than those of a previous ICT kit using crude antigen. We hope that our POCT kit will eventually be promising for supportive diagnosis of symptomatic cysticercosis (particularly NCC cases), both for bedside and field use.

## Supplementary information

*Supplementary Table 1*. The results of Am-ICT and As-ICT kits, with the test-band intensity levels for each serum sample tested. *Supplementary Table 2*. STARD 2015. The supplementary material of this article is available at https://www.parasite-journal.org/10.1051/parasite/2026003/olm.
