# Supplementary material for: Comparative assessment of immunochromatographic test kits using low-molecular-weight antigens from cyst fluids of two different genotypes of Taenia solium for serodiagnosis of human cysticercosis
Source: Parasite. 2026 Jan 23;33:3. doi: 10.1051/parasite/2026003 (PMC12829318; doi:10.1051/parasite/2026003)
Supplement: Supplementary file 1 — Supplementary Table 1. The results of Am-ICT and As-ICT kits, with the test-band intensity levels for each serum sample tested. [file parasite-33-3-s1.pdf]

Supplementary Table 1. The results of Am-ICT and As-ICT kits, with the test-band intensity levels for each serum sample tested, including those from healthy control individuals and individuals with various parasitic infections.

No.	Code	Diagnosis	Suspected countries of infection	Intensity of band in positive cases (level)	
				Am-ICT kit	As-ICT kit
1	Hc1	Healthy person	Thailand	–	–
2	Hc2	Healthy person	Thailand	–	–
3	Hc3	Healthy person	Thailand	–	–
4	Hc4	Healthy person	Thailand	–	–
5	Hc5	Healthy person	Thailand	–	–
6	Hc6	Healthy person	Thailand	–	–
7	Hc7	Healthy person	Thailand	–	–
8	Hc8	Healthy person	Thailand	–	–
9	Hc9	Healthy person	Thailand	–	–
10	Hc10	Healthy person	Thailand	–	–
11	Hc11	Healthy person	Japan	–	–
12	Hc12	Healthy person	Japan	–	–
13	Hc13	Healthy person	Japan	–	–
14	Hc14	Healthy person	Japan	–	–
15	Hc15	Healthy person	Japan	–	–
16	Hc16	Healthy person	Japan	–	–
17	Hc17	Healthy person	Japan	–	–
18	Hc18	Healthy person	Japan	–	–
19	Hc19	Healthy person	Japan	–	–
20	Hc20	Healthy person	Japan	–	–
21	Hc21	Healthy person	Japan	–	–
22	Hc22	Healthy person	Japan	–	–
23	Hc23	Healthy person	Japan	–	–

24	Hc24	Healthy person	Japan	–	–
25	Hc25	Healthy person	Japan	–	–
26	Hc26	Healthy person	Japan	–	–
27	H27	Healthy person	Japan	–	–
28	Hc28	Healthy person	Japan	–	–
29	Hc29	Healthy person	Japan	–	–
30	Hc30	Healthy person	Japan	–	–
31	Cc1	NCC (racemose-type)	China	5	8
32	Cc2	NCC (multiple)	Thailand, Lao PDR, or Madagascar	2	3
33	Cc3	Ocular cysticercosis and taeniasis	Malawi	4	5
34	Cc4	NCC (multiple), *T. solium,* taeniasis	India	4	4
35	Cc5	NCC (multiple, racemose type)	India, Thailand, China or Vietnam	1	3
36	Cc6	NCC (racemose-type)	Japan	2	4
37	Cc7	NCC (multiple), SCC (multiple)	China	2	4
38	Cc8	NCC (multiple), SCC (multiple) and taeniasis	India	3	5
39	Cc9	NCC (multiple), SCC (multiple)	India	3	5
40	Cc10	SCC (solitary) (viable cyst)	Nepal	−	0.5
41	Cc11	NCC (solitary)	India	2	5
42	Cc12	NCC (multiple), SCC (multiple)	Cambodia	4	4
43	Cc13	NCC (multiple)	Nepal	–	–
44	Cc14	NCC (spinal, solitary)	Brazil	5	7
45	Cc15	NCC (multiple), SCC (multiple)	India	–	0.5
46	Cc16	NCC (multiple)	Nepal	–	–
47	Cc17	Ocular cysticercosis	Thailand	2	–
48	Cc18	NCC (multiple), SCC (multiple)	Thailand	2	5
49	Cc19	NCC	Thailand	4	5
50	Cc20	NCC (multiple), SCC (left arm)	Thailand	3	4

51	Cc21	NCC (multiple)	Thailand	4	5
52	Cc22	NCC (multiple)	Thailand	5	6
53	Cc23	NCC (multiple)	Thailand	2	2
54	Cc24	NCC (multiple)	Thailand	3	5
55	Sp1	Sparganosis (proven case)	Thailand	–	–
56	Sp2	Sparganosis (proven case)	Thailand	–	–
57	Sp3	Sparganosis (suspected case), ELISA positive	Thailand	–	–
58	Sp4	Sparganosis (proven case), Histopathology	Thailand	–	–
59	Sp5	Sparganosis (proven case), Histopathology	Thailand	–	–
60	Sp6	Sparganosis (cerebral type, proven case)	Japan	–	–
61	Sp13	Sparganosis and spirometrosis (proven case)	Japan	–	–
62	Sp14	Sparganosis (proven case)	Japan	–	–
63	Sp15	Sparganosis (proven case)	Japan	–	–
64	Sp16	Sparganosis (proven case)	Japan	–	–
65	Sp17	Sparganosis (proven case)	Japan	–	–
66	Sp18	Sparganosis (proven case)	Japan	–	–
67	Ce1	Cystic echinococcosis	unknown	–	–
68	Ce2	Cystic echinococcosis	Peru	–	–
69	Ce3	Cystic echinococcosis	Afganistan	0.5	3
70	Ce4	Cystic echinococcosis	Nepal	–	–
71	Ce5	Cystic echinococcosis	Qinghai, China	–	–
72	Ce6	Cystic echinococcosis	Qinghai, China	–	–
73	Ce7	Cystic echinococcosis	Qinghai, China	–	–
74	Ce8	Cystic echinococcosis	Qinghai, China	–	–
75	Ce9	Cystic echinococcosis	Qinghai, China	–	–
76	Ce10	Cystic echinococcosis	Qinghai, China	–	–
77	Ce11	Cystic echinococcosis	Qinghai, China	–	–

78	Ce12	Cystic echinococcosis	Qinghai, China	0.5	–
79	Ce13	Cystic echinococcosis	Qinghai, China	–	–
80	Ce14	Cystic echinococcosis	Qinghai, China	–	–
81	Ce15	Cystic echinococcosis	Qinghai, China	–	–
82	Ce16	Cystic echinococcosis	Qinghai, China	–	–
83	Ce17	Cystic echinococcosis	Qinghai, China	–	–
84	Ce18	Cystic echinococcosis	Qinghai, China	–	–
85	Ce19	Cystic echinococcosis	Qinghai, China	1	–
86	Ce20	Cystic echinococcosis	Qinghai, China	–	–
87	Ce21	Cystic echinococcosis	Qinghai, China	–	–
88	Ce22	Cystic echinococcosis	Qinghai, China	–	–
89	Ce23	Cystic echinococcosis	Qinghai, China	–	–
90	Ce24	Cystic echinococcosis	Qinghai, China	–	–
91	Ce25	Cystic echinococcosis	Qinghai, China	1	–
92	Ce26	Cystic echinococcosis	Qinghai, China	–	–
93	Ce27	Cystic echinococcosis	Qinghai, China	–	–
94	Ce28	Cystic echinococcosis	Qinghai, China	–	–
95	Ae1	Alveolar echinococcosis	Japan	–	–
96	Ae2	Alveolar echinococcosis	Japan	–	–
97	Ae3	Alveolar echinococcosis	Qinghai, China	–	–
98	Ae4	Alveolar echinococcosis	Qinghai, China	–	–
99	Ae5	Alveolar echinococcosis	Qinghai, China	2	–
100	Ae6	Alveolar echinococcosis	Qinghai, China	–	–
101	Tn1	Taeniasis (*Taenia saginata*)	Thailand	–	–
102	Tn2	Taeniasis (*T. saginata*)	Thailand	–	–
103	Tn3	Taeniasis (*T. saginata*)	Thailand	–	–
104	Tn4	Taeniasis (*T. saginata*)	Thailand	–	–

105	Tn5	Taeniasis (*T. saginata*)	Thailand	–	–
106	Ac1	Angiostrongyliasis (ocular)	Thailand	–	–
107	Ac2	Angiostrongyliasis (ocular)	Thailand	–	–
108	Ac3	Angiostrongyliasis (ocular)	Thailand	–	–
109	Ac4	Angiostrongyliasis (ocular)	Thailand	–	–
110	Ac5	Angiostrongyliasis (ocular)	Thailand	–	–
111	Ac6	Angiostrongyliasis (eosinophilic meningitis) (EITB positive)	Thailand	–	–
112	Ac7	Angiostrongyliasis (eosinophilic meningitis) (EITB positive)	Thailand	–	–
113	Ac8	Angiostrongyliasis (eosinophilic meningitis) (EITB positive)	Thailand	–	–
114	Ac9	Angiostrongyliasis (eosinophilic meningitis) (EITB positive)	Thailand	–	–
115	Ac10	Angiostrongyliasis (eosinophilic meningitis) (EITB positive)	Thailand	–	–
116	Gs1	Gnathostomiasis (ocular)	Thailand	–	–
117	Gs2	Gnathostomiasis (ocular)	Thailand	–	–
118	Gs3	Gnathostomiasis (subcutaneous)	Thailand	–	–
119	Gs4	Gnathostomiasis (ocular)	Thailand	–	–
120	Gs5	Gnathostomiasis (abdominal)	Thailand	–	–
121	Tc1	Toxocariasis (ocular)	Japan	0.5	0.5
122	Tc5	Toxocariasis (visceral)	Japan	–	–
123	Ts2	Trichinosis (proven case)	Thailand	–	–
124	Ts4	Trichinosis (suspected case) (ELISA positive)	Thailand	–	–
125	Ts6	Trichinosis (suspected case) (ELISA positive)	Thailand	–	–
126	Ts7	Trichinosis (suspected case) (ELISA positive)	Thailand	–	–
127	Ts8	Trichinosis (suspected case) (ELISA positive)	Thailand	–	–
128	Fg1	Fascioliasis (*Fasciola gigantica*) (proven case)	Thailand	–	–
129	Fg2	Fascioliasis (*F. gigantica*) (proven case)	Thailand	–	–
130	Fg4	Fascioliasis (*F. gigantica*) (proven case)	Thailand	2	-
131	Fg6	Fascioliasis (*F. gigantica*) (proven case)	Thailand	–	–

132	Fg7	Fascioliasis (*F. gigantica*) (proven case)	Thailand	–	–
133	Fg8	Fascioliasis (*F. gigantica*) (proven case)	Thailand	–	–
134	Fg9	Fascioliasis (*F. gigantica*) (proven case)	Thailand	–	–
135	Fg10	Fascioliasis (*F. gigantica*) (proven case)	Thailand	–	–
136	Fg11	Fascioliasis (*F. gigantica*) (proven case)	Thailand	–	–
137	Fg12	Fascioliasis (*F. gigantica*) (proven case)	Thailand	–	–
138	Ph1	Paragonimiasis (*Paragonimus heterotremus*)	Thailand	–	–
139	Ph2	Paragonimiasis (*P. heterotremus*)	Thailand	–	–
140	Ph3	Paragonimiasis (*P. heterotremus*)	Thailand	–	–
141	Ph5	Paragonimiasis (*P. heterotremus*)	Thailand	–	–
142	Pw 6	Paragonimiasis (Cerebral, due to *Paragonimus westermani*)	Japan	–	–
143	Pa 7	Paragonimiasis (*P. westermani* or *Paragonimus miyazakii*)	Japan	–	–
144	Pa8	Paragonimiasis (*P. westermani* or *P. miyazakii*)	Japan	–	–
145	Pa9	Paragonimiasis (*P. westermani* or *P. miyazakii*)	Japan	–	–
146	Pa10	Paragonimiasis (*P. westermani* or *P. miyazakii*)	Japan	–	–
147	Pa11	Paragonimiasis (*P. westermani* or *P. miyazakii*)	Japan	–	–
148	Am1	Amoebiasis (cerebral abscess)	Japan	–	–
149	Am2	Amoebiasis (liver abscess)	China	–	–
150	Am3	Amoebiasis (liver abscess)	China	–	–
151	Am4	Amoebiasis (liver abscess)	China	–	–
152	Am5	Amoebiasis (liver abscess)	China	–	–
153	Cp-1	Capillariasis (*Capillaria philippinensis*) (proven case)	Japan	–	–
154	Cp-2	Capillariasis (*C. philippinensis*) (proven case)	Japan	–	–
155	Cp-3	Capillariasis (*C. philippinensis*) (proven case)	Thailand	–	–
156	Cp-4	Capillariasis (*C. philippinensis*) (proven case)	Thailand	–	–
157	Cp-5	Capillariasis (*C. philippinensis*) (proven case)	Thailand	–	–
158	Loa Loa-1	Loiasis (proven case)	Japan	3	–

159	Loa Loa-2	Loiasis (proven case)	Japan	0.5	–
160	Ani-1	Anisakiasis (proven case)	Japan	–	–
161	Ani-2	Anisakiasis (proven case)	Japan	–	–
162	Ani-3	Anisakiasis (proven case)	Japan	–	–
163	Ani-4	Anisakiasis (proven case)	Japan	–	–
164	Ani-5	Anisakiasis (proven case)	Japan	–	–

NCC = neurocysticercosis, - = negative. Cystic and alveolar echinococcosis were diagnosed by ECHINOCOCCUS Western Blot IgG kit (LDBIO Diagnostics, Lyon, FRANCE). EITB = Enzyme linked immunoelectrotransfer blot.
